# Antioxidant Treatment Reduces Formation of Structural Cores and Improves Muscle Function in RYR1^Y522S/WT^ Mice

**DOI:** 10.1155/2017/6792694

**Published:** 2017-09-10

**Authors:** Antonio Michelucci, Alessandro De Marco, Flavia A. Guarnier, Feliciano Protasi, Simona Boncompagni

**Affiliations:** ^1^Center for Research on Aging and Translational Medicine (CeSI-MeT), University G. d'Annunzio of Chieti, 66100 Chieti, Italy; ^2^Department of Neuroscience, Imaging, and Clinical Sciences (DNICS), University G. d'Annunzio of Chieti, 66100 Chieti, Italy; ^3^Department of General Pathology, University Estadual de Londrina, 86057-970 Londrina, PR, Brazil; ^4^Department of Medicine and Aging Science (DMSI), University G. d'Annunzio of Chieti, 66100 Chieti, Italy

## Abstract

Central core disease (CCD) is a congenital myopathy linked to mutations in the ryanodine receptor type 1 (RYR1), the sarcoplasmic reticulum Ca^2+^ release channel of skeletal muscle. CCD is characterized by formation of amorphous *cores* within muscle fibers, lacking mitochondrial activity. In skeletal muscle of RYR1^Y522S/WT^ knock-in mice, carrying a human mutation in RYR1 linked to malignant hyperthermia (MH) with *cores*, oxidative stress is elevated and fibers present severe mitochondrial damage and *cores*. We treated RYR1^Y522S/WT^ mice with N-acetylcysteine (NAC), an antioxidant provided *ad libitum* in drinking water for either 2 or 6 months. Our results show that 2 months of NAC treatment starting at 2 months of age, when mitochondrial and fiber damage was still minimal, (i) reduce formation of *unstructured* and *contracture cores*, (ii) improve muscle function, and (iii) decrease mitochondrial damage. The beneficial effect of NAC treatment is also evident following 6 months of treatment starting at 4 months of age, when structural damage was at an advanced stage. NAC exerts its protective effect likely by lowering oxidative stress, as supported by the reduction of 3-NT and SOD2 levels. This work suggests that NAC administration is beneficial to prevent mitochondrial damage and formation of *cores* and improve muscle function in RYR1^Y522S/WT^ mice.

## 1. Introduction

Central core disease (CCD), one of the most common human congenital myopathies, is an inherited neuromuscular disorder characterized by hypotonia and proximal muscle weakness, which cause motor developmental delay in children [1, 2]. Diagnosis of CCD is confirmed by histological examination of muscle biopsies showing amorphous central areas (i.e., *cores*) lacking glycolytic/oxidative enzymes and mitochondria [3]. Disorganization of contractile and sarcotubular systems is also typical in *cores* [4]. To date, management of patients/children is essentially supportive and based on physiotherapic approaches and no curative treatments are available. Hence, a deeper understanding of the molecular mechanisms underlying mitochondrial damage and formation of *cores* in CCD is needed to develop effective therapeutic interventions.

In humans, about 90% of CCD cases are linked to mutations in the ryanodine receptor type 1 (RYR1) gene [5], encoding for the sarcoplasmic reticulum (SR) Ca^2+^ release channel of skeletal muscle. RYR1 is a large protein of about 2200 kDa specifically localized in calcium release units (CRUs), the intracellular junctions formed by the close apposition of transverse-tubules (TT) to the SR. RYR1 in CRUs is part of macromolecular complex deputed to excitation-contraction (EC) coupling, the mechanism that allows transduction of the action potential into Ca^2+^ release from the SR [6, 7]. Mutations in RYR1 gene, which causes abnormalities in the opening probability of the Ca^2+^ channel, are also often associated to malignant hyperthermia (MH) susceptibility, an inherited pharmacogenetic subclinical myopathy, characterized by a life-threatening hypermetabolic response to commonly used halogenated/volatile anesthetics (i.e., halothane, isofluorane) [8, 9]. An association between CCD and MH exists as individuals with MH may have muscle biopsies with *cores* [10, 11], while CCD patients may be at risk for hyperthermic episodes during anesthetic procedures, as also confirmed by *in vitro* caffeine-halothane contracture testing (either IVCT, *in vitro* contracture test, or the CHCT, caffeine-halothane contracture test), performed on muscle biopsies [2, 12–14].

In the 2006, an animal model (RYR1-Y522S knock-in mice) carrying a human gain-of-function mutation associated to MH, skeletal muscle weakness, and formation of *cores* was generated and characterized [10]. Heterozygous Y522S mice (RYR1^Y522S/WT^) suffer lethal MH crises when acutely exposed to both anesthetics and heat [15, 16] and develop structural *cores* [17]. Muscles of RYR1^Y522S/WT^ mice also exhibit a marked temperature-dependent increase in resting myoplasmic Ca^2+^, excessive oxidative stress [16], and enhanced mitochondrial superoxide flashes activity [18]. The current view of the molecular mechanisms underlying the phenotype of RYR1^Y522S/WT^ mice is that the Y522S mutation promotes a significant increase of the opening probability of the RYR1 channel, which causes SR Ca^2+^ leak and overproduction of reactive oxygen and nitrogen species (ROS and RNS), as a consequence of the increased Ca^2+^-dependent mitochondrial activity. In turn, excessive ROS and RNS would determine nitrosylation/glutathionylation of RYR1, oxidative modifications responsible of further increase of RYR1 opening probability [16]. The feed-forward mechanism triggered by ROS/RNS would in principle play a pivotal role: (i) acutely, in anesthetic- and heat-induced lethal MH episodes and (ii) chronically, in mitochondrial damage and development of *cores*, two types of structural alterations that resemble CCD in humans [17].

Here, we treated RYR1^Y522S/WT^ mice with N-acetylcysteine (NAC), a potent antioxidant provided *ad libitum* in drinking water (1% *w*/*v*) for either 2 months (2–4 months of age, starting when mitochondrial and fiber damage was still minimal) or 6 months (4–10 months of age; starting when structural damage was already at an advanced stage) [17], and evaluated the effect of this pharmacological treatment on formation of *cores*, muscle function, mitochondrial damage, and levels of oxidative stress in *extensor digitorum longus* (EDL) muscles. The results collected in this study indicate that NAC administration is beneficial to prevent/reduce mitochondrial damage and formation of *cores* and improve muscle function in RYR1^Y522S/WT^ mice.

## 2. Materials and Methods

### 2.1. RYR1^Y522S/WT^ Mice

Heterozygous Y522S mice were generated as previously described [15]. Mice were housed in microisolator cages at 20°C in a 12 hr light/dark cycle and provided free access to water and food. Animals used in this study were all males, and analyses were carried out in EDL muscles. All experiments were conducted according to the Directive of the European Union 2010/63/UE, and animal protocols were approved by the Committee on the Ethics of Animal Experiments of the University of Chieti (15/2011/CEISA/COM). All surgeries were made to minimize animal suffering; animals were anesthetized and then sacrificed by cervical dislocation.

### 2.2. NAC Treatment

RYR1^Y522S/WT^ mice were randomly assigned to the experimental groups: untreated or NAC-treated RYR1^Y522S/WT^ mice. NAC (Sigma-Aldrich, Milan, Italy) was provided *in vivo* in drinking water at the final concentration of 1% weight/volume (1% *w*/*v*) as previously described [19] for either 2 months (from 2–4 months of age) or 6 months (from 4–10 months of age).

### 2.3. Preparation and Analysis of Samples in Light and Electron Microscopy (EM)

EDL muscles were dissected, fixed at room temperature with 3.5% glutaraldehyde in 0.1 M NaCaCo buffer (pH 7.2), and kept at 4°C in fixative until further use. Small bundles of fixed muscles were then postfixed, embedded, stained en-block, and sectioned as previously described [17, 20]. For EM, ultrathin sections (~50 nm) were examined after staining in 4% uranyl acetate and lead citrate, with a Morgagni Series 268D electron microscope (FEI Company, Brno, Czech Republic), equipped with Megaview III digital camera (Olympus Soft Imaging Solutions GmbH, Munster, Germany) at 60 kV. For histology, 700 nm thick sections were stained in a solution containing 1% Toluidine blue O and 1% sodium borate tetra in distilled water for 3 min on a hot plate at 55–60°C. After washing and drying, sections were finally mounted with mounting medium (DPX Mountant for histology, Sigma-Aldrich, Milan, Italy).

### 2.4. Quantitative Analysis in Histology and EM Preparations

Consider the following:

1. Histological sections were examined under direct illumination and/or phase contrast optics with a Leica DMLB fluorescence microscope (Leica Microsystem, Vienna, Austria), and individual EDL fibers were visually scored for the presence of either *unstructured* or *contracture cores* as in [17].
2. Number/area, size, and volume of mitochondria (and number/area of CRUs and mitochondria CRU pairs) were determined in EDL fibers as follows: (a) Mitochondrial volume was determined using the well-established stereology point-counting technique [21, 22] in micrographs taken from transversal sections at magnification 7100x. Briefly, after superimposing an orthogonal array of dots at a spacing of 0.20 *μ*m to the electron micrographs, ratio between numbers of dots falling within mitochondrial profiles and total number of dots covering the whole image was used to calculate the relative fiber volume occupied by mitochondria. (b) Number of severely damaged mitochondria was counted in longitudinal sections, and their frequency was reported as an average number in 100 *μ*m^2^ and as % of mitochondria evaluated. Mitochondria were classified as severely damaged as previously described [17]. (c) Average mitochondrial size (nm^2^) of apparently normal mitochondria was measured in longitudinal sections using the analysis software of the EM digital camera (Olympus Soft Imaging Solutions GmbH, Munster, Germany). Severely damaged mitochondria, included in (b), were excluded from this analysis. (d) Number/area of CRUs, mitochondria, and mitochondria CRU pairs was also evaluated in longitudinal sections and reported as the average number in 100 *μ*m^2^.

### 2.5. Immunolabeling and Confocal Microscopy (CM)

EDL muscles were dissected from sacrificed mice and fixed with paraformaldehyde 2% for 1-2 hrs at room temperature. Small bundles of muscles were processed for double immunostaining as previously described [23]. Primary antibodies used (a) mouse monoclonal anti-RyR1/RyR3 34C (1 : 20) (Developmental Studies Hybridoma Bank, University of Iowa, Iowa, USA) and (b) rabbit polyclonal antimitochondrial preprotein translocases of the outer membrane, TOM20 (1 : 50) (Santa Cruz Biotechnology, Inc., Dallas, TX, USA). Secondary antibodies used (a) Cy5-labeled goat anti-mouse IgG and (b) Cy3-labeled goat anti-rabbit IgG (Jackson ImmunoResearch Laboratories, West Grove, PA, USA). Images were acquired using a Zeiss LSM510 META laser-scanning confocal microscope system (Zeiss, Jena, Germany) equipped with Zeiss Axiovert 200 inverted microscope and a Plan Neofluar oil-immersion objective (63X/1.3 NA). Negative controls for each immunostaining assay were performed by immunolabeling of samples with only secondary antibodies.

### 2.6. Quantitative Plasma and Serum Analyses

Blood levels of creatine kinase (CK) and lactate dehydrogenase (LDH), markers of fiber damage, were spectrophotometrically measured in serum (CK) and plasma (LDH) samples obtained from mice as previously described [19], by using a Screen Touch Master spectrophotometer (Hospitex Diagnostic, Sesto Fiorentino, Italy).

### 2.7. Calpain Activity

The activity of calpain was measured in total hind limb muscle homogenates, by a chemiluminescence assay using a Calpain Protease Assay kit (Calpain-Glo Protease Assay®, Promega; Madison, WI, USA). The assay provides a proluminescent calpain substrate, in a buffer system optimized for calpain and luciferase activities. During the assay, calpain cleavage of the substrate generates a *glow-type* luminescent signal produced by the luciferase reaction. In this homogeneous, coupled-enzyme format, the signal is proportional to the amount of calpain activity present in the sample [24]. In this study, total hind limb muscle homogenates were prepared at a concentration of 6.25 mg/mL in 10 mM KH_2_PO_4_ buffer, pH 7.4 in 0.9% NaCl and finally processed according to the manufacturer's instructions. Results are expressed as calpain activity/mg of muscle tissue.

### 2.8. Carbonyl Protein Content

Protein carbonyl group formations are classic and immediate biomarkers of oxidative modification to proteins, which may promote disorganization of contractile structures [25, 26]. 2,4-Dinitrophenylhydrazine (DNPH) tagging of protein carbonyls has been one of the most common measures of oxidative stress and consequent protein damage. Carbonyl protein content was measured as previously described [25], with modifications. Briefly, a mix of total hind limb muscles (50 mg/mL) was homogenized in 50 mM phosphate buffer, 1 mM ethylenediamine tetraacetic acid, pH 7.4, and tissue samples were centrifuged at 600*g*/10 min/4°C. A volume of 200 *μ*L of DNPH was added to 200 *μ*L of supernatant and incubated at room temperature. After a 30 min incubation, 100% trichloroacetic acid (TCA) was added and samples were placed on ice for 5 min and then spinned at maximal speed for 2 min. Supernatants were discarded without disturbing pellets, which were washed in cold acetone and placed at −20°C for 5 min. Then, acetone was carefully removed, and pellets were dissolved in 0.5 mL 6 M guanidine hydrochloride to be read at 375 nm. To calculate the protein carbonyl content, the following formula was used: C = [(OD 375 nm)/6.364 × (100)] nmol/well, where 6.364 is the extinction coefficient using the enclosed 96-well plates in mM (=22 mM-1 cm-1 × 0.2893 cm path length in well). Results were expressed as nmol carbonyl/mg of total protein, which were quantified in each sample at 280 nm.

### 2.9. Western Blot Analyses

For assessment of 3-nitrotyrosyne (3-NT) content, mixed muscles from hind limb were homogenized on ice in a buffer containing 50 mM Tris-HCl, pH 7.4; 1% NP-40; 0.25% sodium deoxycholate; 150 mM NaCl; 1 mM EDTA; 1 mM PMSF; protease inhibitors. After centrifugation at 10000*g* for 15 min at 4°C, supernatants were collected and total protein concentration was determined using Bio-Rad Protein assay (Bio-Rad laboratories, CA). 40 *μ*g of total proteins was separated by SDS-PAGE in a 10% polyacrylamide gel, followed by western blotting using anti 3-NT mouse monoclonal antibody (1 : 500, Merk Millipore, Milan, Italy) and a horseradish peroxidase- (HRP-) conjugated anti-mouse secondary antibody (Merck Millipore, Darmstadt, Germany). Visualization and densitometric quantification of signals were done using the imaging system Alliance Mini 4 with Alliance 1D MAX software (UVItec, Cambridge, UK). After measuring 3-NT, the membranes were stripped by Tris/SDS buffer with 2-mercaptoethanol. After blocking, the membranes were incubated with anti-glyceraldehyde-3-phosphate dehydrogenase (GAPDH) antibody (OriGene, Rockville, MD, USA) for normalization to the protein content within each band.

For assessment of Cu/Zn-superoxide dismutase (SOD1) and Mn-superoxide dismutase (SOD2) protein levels, western blot experiments were performed as follows: mixed muscles from hind limb were homogenized on ice in RIPA buffer (50 mM Tris-HCl, pH 8.0; 150 mM NaCl; 0.5% sodium deoxycholate; 0.1% SDS; 1% NP-40; 0.1 mM PMSF; protease inhibitors). Homogenates were centrifuged at 10000*g* for 15 min at 4°C, supernatants were collected, and total protein concentration was determined using Bio-Rad Protein assay (Bio-Rad laboratories, Hercules, CA, USA). Protein samples (5 *μ*g), solubilized in 2× sample buffer (125 mM Tris-HCl, pH 6.8; 4% SDS; 20% glycerol; 0.004% bromophenol blue; 10% 2-mercaptoethanol), were loaded on a 12% acrylamide gel, separated by SDS-PAGE, and transferred to nitrocellulose membrane. Membranes were probed using primary antibodies against SOD1 (1 : 1000, Santa Cruz Biotechnology, Dallas, TX, USA), SOD2 (1 : 2000, Santa Cruz Biotechnology, Dallas, TX, USA), and GAPDH (1 : 10000, OriGene, Rockville, MD, USA) overnight at 4°C. HRP-conjugated anti-mouse or rabbit (1 : 10000, Merck Millipore, Darmstadt, Germany) was used as a secondary antibody, and peroxidase activity was detected using an enhanced chemiluminescence (ECL) kit (Perkin Elmer, Waltham, MA, USA). The bands were visualized, and densitometric quantification of signals was performed using the imaging system Alliance Mini 4 with Alliance 1D MAX software (UVItec, Cambridge, UK).

### 2.10. Grip Strength Test

Strength developed by mice during instinctive grasp was measured as previously described [27]. Briefly, mice were held by the tail and allowed to firmly grasped to a metal grating, connected to the shaft of a Shimpo Fgv 0.5X force transducer (Metrotec Group, Lezo, Spain), with fore and hind limbs before a gentle pull was exerted on the tail. Measurements of peak force generated by each mouse were repeated three times with appropriate intervals (at least 30 sec) to avoid fatigue, and the highest value of peak force (normalized to total body mass) measured before each experiment was used.

### 2.11. Force and Contraction Kinetics of Intact EDL Muscles

EDL muscles were dissected from WT, untreated RYR1^Y522S/WT^, and NAC-treated RYR1^Y522S/WT^ mice and placed in a dish containing Krebs solution with the following composition in mM: 118 NaCl, 4.7 KCl, 1.2 MgSO_4_, 2.5 CaCl_2_, 1.2 KH_2_PO_4_, 25 NaHCO_3_, and 11 glucose. Individual EDLs were then pinned, tied with fine silk sutures at each end, and mounted vertically between two platinum electrodes immersed in an organ chamber filled with Krebs solution and attached to a servo motor and force transducer (model 1200A, Aurora Scientific, ON, Canada). Temperature was kept between 23–25°C. Before starting the experimental protocol, stimulation level and optimal muscle length (*L_0_*) were determined using a series of 80 Hz-tetani in order to stretch the muscle to the length that generated maximal force (*F_0_*). After optimization of the stimulation conditions, EDL muscles were subjected to a force-frequency protocol based on a series of train pulses of 500 ms duration each as follows (in Hz): 1, 5, 10, 20, 40, 60, 80, 100, 120, and 140. After 5 min at rest, the same EDL muscles were subjected to a single-sustained high frequency tetanus (120 Hz, 2 sec). Muscle force was recorded using a dynamic muscle control (DMC) software and analyzed using dynamic muscle analysis (DMA) software (both from: Aurora Scientific, ON, Canada). Specific force (mN/mm^2^) was calculated by normalizing the absolute force (mN) to the cross sectional area (CSA, mm^2^) obtained as the following: muscle wet weight (mg)/*L_0_* (mm)∗1.06 (mg/mm^3^).

### 2.12. Statistical Analyses

Statistical significance for the quantitative analysis of fibers presenting structural alterations (i.e., *unstructured* and *contracture cores*) was evaluated using two-tailed Fisher's exact test. One-way ANOVA followed by post hoc Tukey test was used for statistical analyses of all other experiments except for those regarding the time courses of *in vivo* grip strength and the force-frequency of intact EDL muscles, in which repeated measures ANOVA was used followed by post hoc Tukey test for the pairwise comparisons. In all cases, differences were considered statistically significant at *p* < 0.05. Two-tailed Fisher's exact tests were performed using GraphPad software, whereas one-way ANOVA and repeated measures ANOVA were performed using Origin 8.0 software.

## 3. Results

The effect of NAC treatment on structure, function, and oxidative stress levels of EDL muscles from RYR1^Y522S/WT^ mice was evaluated at (a) 4 months of age after 2 months of treatment (starting when mitochondrial and fiber damage was still minimal) and at (b) 10 months of age after 6 months of treatment (starting when mitochondrial damage was already at an advanced stage) [17]. In the manuscript, we will refer to 2 and 6 months of treatment as *short-term* and *long-term* NAC treatments, respectively.

### 3.1. NAC Treatment Reduces Formation of Structural Cores, Fiber Damage, and Proteolytic Degradation in EDL Muscles of RYR1^Y522S/WT^ Mice

**Figure 1:**
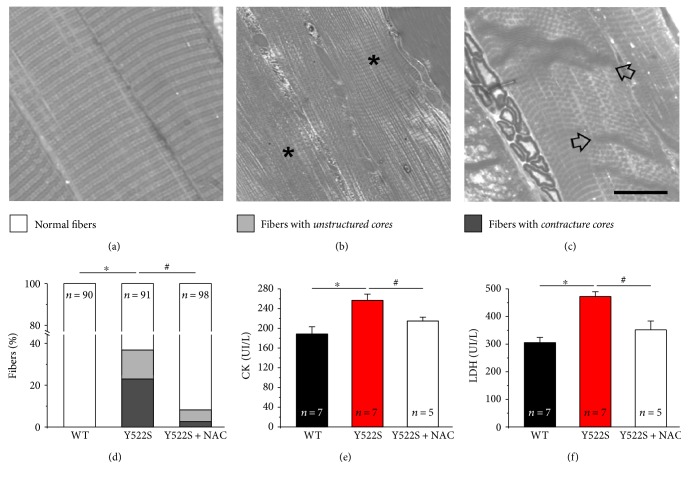
Structural *cores* and blood markers of muscle damage (CK and LDH) at 4 months of age. (a–c) Representative histological images of normal fibers (a), fibers with *unstructured cores* (b), and fibers with *contracture cores* (c). (d) Percentage of EDL fibers presenting the features classified in (a–c) (white: *normal fibers*; grey: *fibers with unstructured cores*; and dark grey: *fibers with contracture cores*). See also Table S1. (e and f) Serum levels of creatine kinase (CK) and lactate dehydrogenase (LDH). In (e) and (f), data are given as mean ± SEM; ^∗^*p* < 0.05, WT versus RYR1^Y522S/WT^ mice; ^#^*p* < 0.05, untreated RYR1^Y522S/WT^ versus NAC-treated RYR1^Y522S/WT^ mice. In (d), *n* = number of fibers analyzed; in (e-f), *n* = number of mice. *Scale bar: 10 μm*.

Skeletal fibers from RYR1^Y522S/WT^ mice develop *unstructured* and *contracture cores* [17]. To evaluate the effect of NAC in reducing formation of *cores*, using histological sections, we analyzed and classified fibers in three different categories, as previously described [17]: (a) *normal fibers*, presenting a well-preserved cross striation pattern (Figure 1(a)); (b) fibers with *unstructured cores*, presenting extensive areas lacking cross striation (Figure 1(b), asterisks); and (c) fibers with *contracture cores*, exhibiting areas of extreme sarcomere shortening (Figure 1(c), arrows). Quantitative analysis of the relative percentage of fibers, presenting the different features, indicates that NAC treatment was effective in preventing the formation of structural *cores* (Figure 1(d) and Table S1 available online at https://doi.org/10.1155/2017/6792694). Specifically, *unstructured* and *contracture cores*, which are not present in WT fibers, were found, respectively, in 14% and 23% of fibers in RYR1^Y522S/WT^ EDL muscles (Figure 1(d)). However, following NAC treatment, the number of RYR1^Y522S/WT^ EDL fibers containing *unstructured* and *contracture cores* was significantly reduced to 6% and 3%, respectively (Figure 1(d)). The decrease in the number of fibers containing *cores* results in a parallel increase in the percentage of normal fibers (from 63 to 92%). Data plotted in Figure 1 are available in Table S1.

As fiber damage results in changes in blood parameters, we performed biochemical measurements of markers of muscle damage in blood samples. Specifically, we evaluated serum and plasma levels of the muscle-specific isoforms of creatine kinase (CK, in serum) and lactate dehydrogenase (LDH, in plasma) [28]. Consistent with the higher percentage of fibers presenting structural alterations, in RYR1^Y522S/WT^ samples, both CK and LDH levels were about ~30% higher than those in aged-matched WT mice (Figures 1(e) and 1(f)). The protective effect of NAC was confirmed by these experiments: 2 months of NAC treatment was effective in reducing the amount of both CK and LDH in blood samples from RYR1^Y522S/WT^ mice to values closer to that of WT (Figures 1(e) and 1(f)), a result consistent with the significant reduction in the incidence of fibers presenting *unstructured* and *contracture cores* (Figure 1(d)).

**Figure 2:**
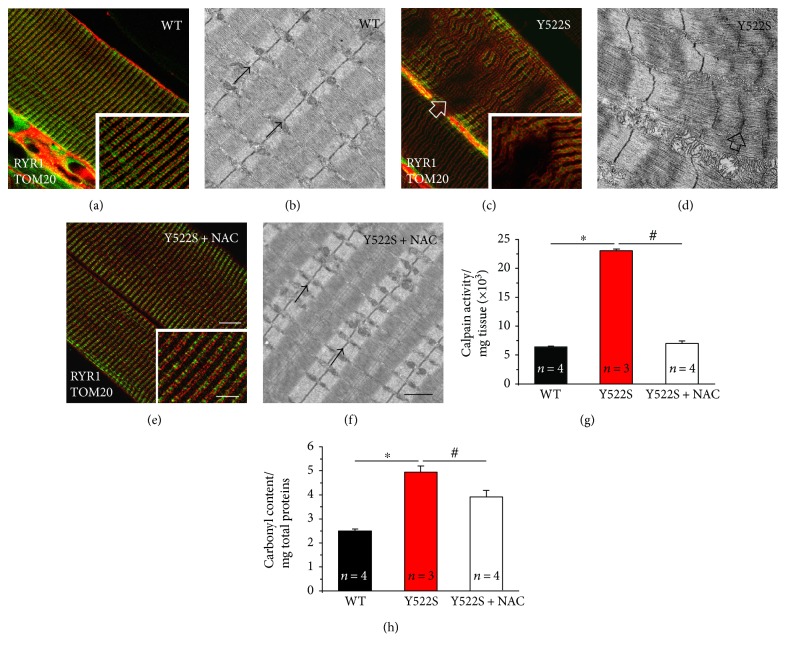
Fiber ultrastructure, calpain activity, and carbonyl protein content at 4 months of age. (a–f) Representative immunofluorescence and EM images showing fluorescent cross striation (a, c, and e) and myofibrillar/sarcomeric organization (b, d, and f) in EDL fibers. In (b) and (f), small arrows point at *Z*-lines, while in (c and d) large arrows point to areas in which cross striation and sarcomeric structure are compromised. (g) Calpain activity expressed in total hind limb muscle homogenates. (h) Carbonyl protein content in EDL muscle homogenates. In (g and h), data are given as mean ± SEM; ^∗^*p* < 0.05, WT versus RYR1^Y522S/WT^ mice; ^#^*p* < 0.05, untreated RYR1^Y522S/WT^ versus NAC-treated RYR1^Y522S/WT^ mice. In (g and h), *n* = number of mice. *Scale bars* in (a, c, and e) 10 *μ*m (insets 5 *μ*m); in (b, d, and f) 1 *μ*m.

We also analyzed EDL muscles using a combination of immunofluorescence for CM and EM and assessed proteolytic degradation by measuring calpain activity and carbonyl protein content (Figure 2). In adult EDL fibers from WT mice, both CRUs, marked with an antibody that recognized RYR1, and mitochondria, marked with an antibody that labels translocase of outer mitochondrial membrane 20 homolog (TOM20), form double rows of cross striation (Figure 2(a) and inset). This fluorescence pattern is consistent with positioning of both CRUs and mitochondria at the I band, on either side of the *Z*-line (pointed by arrows in Figures 2(b) and 2(f); see [20] for additional detail on the specific disposition of CRUs and mitochondria in adult skeletal fibers). In RYR1^Y522S/WT^ EDL fibers, this precise cross striation pattern was often compromised (arrow in Figure 2(c) and inset). These areas lacking staining (pointed by arrows in Figure 1(c)) reflect the presence of *contracture cores*, visible in EM as regions with shortened sarcomeres (arrow in Figure 2(d)). NAC treatment in RYR1^Y522S/WT^ mice restored the cross striation pattern in the large majority of fibers (consistent with reduction of fibers with *cores*; Figure 1(d)): both red and green staining were perfectly transversal (Figure 2(e)), while ultrastructure of myofibrils and sarcomeres at the EM examination was virtually indistinguishable from that of WT fibers (compare Figures 2(b) and 2(f)).

The RYR1-Y522S mutation causes leak of Ca^2+^ from the SR [16]. As excessive myoplasmic Ca^2+^ concentration activates calpains that cleave a variety of substrates, including myofibrillar proteins [24, 29, 30], calpain-mediated degradation could contribute to the ultrastructural alterations observed in RYR1^Y522S/WT^ muscle fibers. We measured the total calpain activity, which was markedly elevated in muscles from RYR1^Y522S/WT^ compared to that from WT (Figure 2(g)), but lowered to levels very similar to those from WT following NAC treatment (Figure 2(g)). In addition, we also evaluated the total carbonyl protein content in the same muscle homogenates, an important biomarkers of oxidative modification of proteins [25, 26]. Also in this case, the total amount of carbonyl proteins, which was abnormally elevated in RYR1^Y522S/WT^, was lowered of about 20% by NAC treatment, although it remained still higher than that in WT (Figure 2(h)).

### 3.2. NAC Treatment Improves *In Vivo* Grip Strength and Ex Vivo Muscle Contractile Function in RYR1^Y522S/WT^ Mice

**Figure 3:**
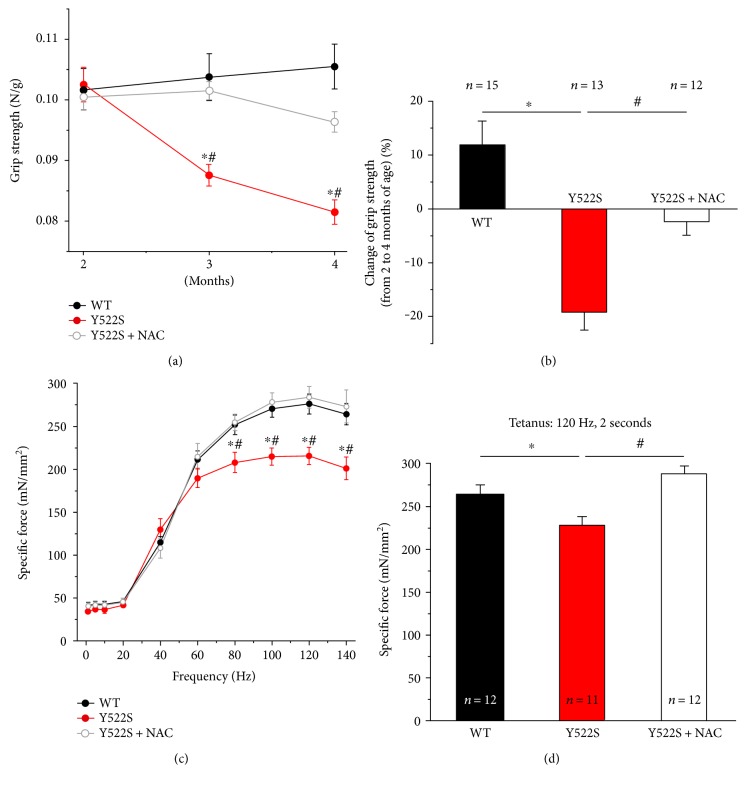
*In vivo* grip strength and ex vivo specific force at 4 months of age. (a) Time-course of grip strength from 2 (beginning of short-term NAC treatment) to 4 months (end of short-term NAC treatment) of age expressed as force on body weight (N/g). (b) Change in grip strength from 2 to 4 months of age (shown as a percentage). (c and d) Force-frequency (1–140 Hz) relationship curves of specific force (c) and specific force during a single 2 s, 120 Hz stimulation train (d) recorded for the same EDL muscles. Data are given as mean ± SEM; ^∗^*p* < 0.05, WT versus RYR1^Y522S/WT^ mice; ^#^*p* < 0.05, untreated RYR1^Y522S/WT^ versus NAC-treated RYR1^Y522S/WT^ mice. In (b), *n* = number of mice; in (d), *n* = number of EDL muscles.

As NAC treatment was very effective in protecting muscle fibers of RYR1^Y522S/WT^ mice from structural damage, we performed *in vivo* grip strength test and ex vivo contractile experiments on intact EDL muscles, to evaluate whether NAC was also able to improve muscle function of RYR1^Y522S/WT^ knock-in mice. Grip strength was evaluated at 3 different time points between 2 (beginning of the *short-term* NAC treatment) and 4 months of age (end of *short-term* NAC treatment). Results of these evaluations of force are plotted in Figures 3(a) and 3(b). While no significant difference was found among the three groups of mice tested at 2 months of age, in the following 2 months, (a) WT mice showed a small, but progressive, rise in grip strength of about 10%; (b) untreated RYR1^Y522S/WT^ mice exhibited a pronounced decay, with a force reduction of about 20%; and finally (c) NAC-treated RYR1^Y522S/WT^ mice displayed an ameliorated muscle function compared to untreated RYR1^Y522S/WT^ mice, with a reduction in grasp force of only 4%. To verify if NAC treatment directly improved muscle function, we evaluated ex vivo force-frequency and 2-second tetanic force in isolated EDL muscles at 4 months of age using an *in vitro* setting (Figures 3(c) and 3(d)). The force-frequency relationship curves during high-frequency stimulation (from 80 Hz to 140 Hz) plotted in Figure 3(c) showed that EDL muscles from RYR1^Y522S/WT^ mice developed a specific force (mN/mm^2^) significantly lower than that from WT, force that was efficiently rescued by NAC treatment. Interestingly, the percentage of relative force, normalized to the maximum (*F*_0_), for the half-maximal frequency (Hz_60_) was significantly higher in RYR1^Y522S/WT^ muscles (87.7 ± 2.8) than that in the WT (74.8 ± 7.4) and NAC-treated RYR1^Y522S/WT^ (77.0 ± 5.6) muscles (Supplementary Figure S2 A). We also evaluated the maximal force developed by the same EDL muscles by applying a single train stimulus (120 Hz for 2 seconds) to generate a fused tetanus (Figure 3(d)). EDL muscles from RYR1^Y522S/WT^ mice exhibited a maximal specific tetanic force significantly lower to that developed by EDL muscles from WT mice (227.2 ± 11.6 versus 264.0 ± 10.6 mN/mm^2^); while following two months of NAC treatment, maximal specific tetanic force expressed by EDL muscles from RYR1^Y522S/WT^ mice was rescued to values similar to those of WT muscles (287.7 ± 8.8 mN/mm^2^) (Figure 3(d)).

### 3.3. NAC Treatment Reduces Mitochondrial Swelling and Damage in EDL Muscle of RYR1^Y522S/WT^ Mice

**Figure 4:**
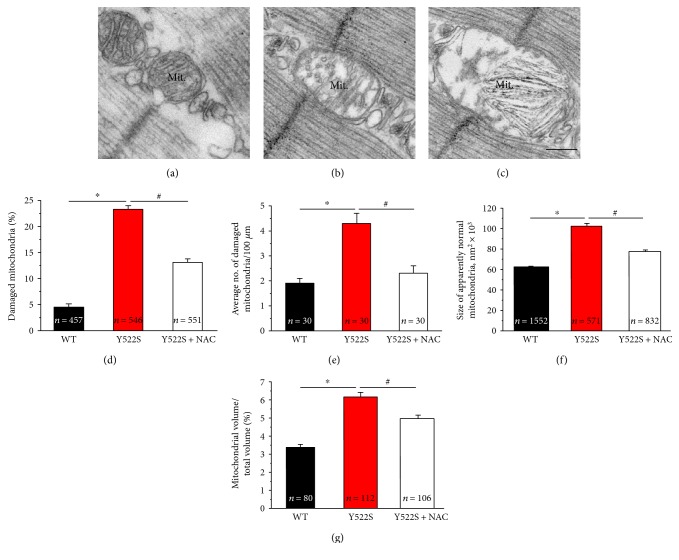
Mitochondrial damage, size, and volume at 4 months of age. (a–c) Representative EM images displaying apparently normal (a) and damaged mitochondria (b and c). (d) Percentage of damaged mitochondria. (e) Average number of damaged mitochondria/area of EM section. (f) Average size of apparently normal mitochondria (i.e., mitochondria not included in the quantitative analysis of (d and e)). (g) Percentage of fiber volume occupied by mitochondria. See also Table S2. Data are given as mean ± SEM; ^∗^*p* < 0.05, WT versus RYR1^Y522S/WT^ mice; ^#^*p* < 0.05, untreated RYR1^Y522S/WT^ versus NAC-treated RYR1^Y522S/WT^ mice. In (d and f), *n* = number of measurements; in (e and g), *n* = number of fibers analyzed. *Scale bar:* 0.1 *μ*m.

As previously reported, mitochondrial damage underlies formation of *cores* in RYR1^Y522S/WT^ mice [17]. Here, we confirmed previous findings: (a) damaged mitochondria (such as those in Figures 4(b) and 4(c)) were significantly more frequent in EDL fibers from RYR1^Y522S/WT^ mice than in WT (Figures 4(d) and 4(e)); (b) also, mitochondria that are apparently normal (dark appearance, such as that in Figure 4(a)) were larger in size, suggesting that the organelles are swollen (Figure 4(f)); (c) total mitochondrial volume was increased in fibers from RYR1^Y522S/WT^ (Figure 4(g)). *Short-term* NAC treatment significantly rescued all these features: (a) the number of mitochondria presenting structural damage was decreased in fibers from mice treated with NAC (Figures 4(d) and 4(e)); (b) treatment of RYR1^Y522S/WT^ mice with NAC was also effective in reducing the size of apparently normal mitochondria and the relative fiber volume occupied by these organelles (Figures 4(f) and 4(g)). Data plotted in Figures 4(d), 4(e), 4(f), and 4(g) are reported in Table S2.

As in adult skeletal muscle, mitochondria are usually closely associated with CRUs [20]; here, we verified whether NAC exert a beneficial effect on the CRU-mitochondrial interaction. We evaluated the frequency of mitochondria, CRUs, and mitochondria-CRU pairs (Supplementary Figure S1): (a) number/area of both mitochondria and CRUs is decreased in RYR1^Y522S/WT^ compared to that in WT (Supplementary Figure S1 B and C), which in turn causes a great reduction in the number/area of mitochondria-CRU pairs (Supplementary Figure S1 D). Treatment with NAC was able to partially rescue the number/area of mitochondria (Supplementary Figure S1 B) and of mitochondria-CRU pairs (Supplementary Figure S1 D). However, NAC was not able to rescue the number/area of CRUs (Supplementary Figure S1 C). Data plotted in Supplementary Figure S1 B-C are reported in Table S3.

### 3.4. NAC Was Effective in Preventing Decay of Muscle Structure/Function in RYR1^Y522S/WT^ Mice Also during Long-Term Treatment

**Figure 5:**
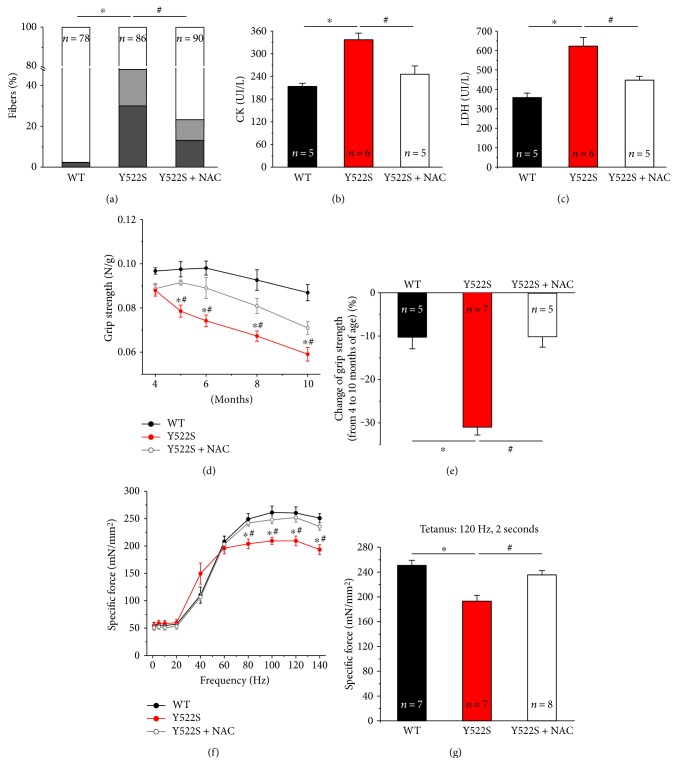
Effects of *long-term* NAC treatment at 10 months of age. (a) Quantitative analysis of EDL fibers presenting the features classified in Figures 1(a), 1(b), and 1(c) shown as percentage of fibers analyzed (white: *normal fibers*; grey: *fibers with unstructured cores*; and dark grey: *fibers with contracture cores*). See also Table S4. (b and c) Serum levels of creatine kinase (CK) and lactate dehydrogenase (LDH). (d) Time course of grip strength from 4 (beginning of *long-term* NAC treatment) to 10 months (end of *long-term* NAC treatment) of age expressed as force on body weight (N/g). (e) Change in grip strength from 4 to 10 months of age (shown as a percentage). (f and g) Force-frequency (1–140 Hz) relationship curves of specific force (f) and specific force during a single 2 s, 120 Hz stimulation train (g) recorded for the same EDL muscles. In s (b–g), data are given as mean ± SEM; ^∗^*p* < 0.05, WT versus RYR1^Y522S/WT^ mice; ^#^*p* < 0.05, untreated RYR1^Y522S/WT^ versus NAC-treated RYR1^Y522S/WT^ mice. In (a), *n* = number of EDL fibers analyzed; in (b–e), *n* = number of mice; in (f and g), *n* = number of EDL muscles.

We have previously shown that fiber damage in RYR1^Y522S/WT^ mice becomes quite severe with increasing age [17]. Here, we tested the *long-term* efficacy of NAC in reducing/preventing structural and functional decay of RYR1^Y522S/WT^ fibers by treating mice for 6 months with NAC, starting at 4 months of age when structural damage was already at an advanced stage. Results of these experiments are shown in Figure 5: (a) the percentage of fibers presenting *unstructured* and *contracture cores* (resp., 18% and 30% in RYR1^Y522S/WT^ mice) was significantly reduced (10% and 13%, resp.) by NAC administration (Figure 5(a)). Data plotted in Figure 5(a) are reported in Table S4. As done in samples from 4-month-old mice treated for 2 months, we also measured blood levels of CK and LDH at 10 months of age following 6 months of NAC treatment: both CK and LDH were significantly elevated in RYR1^Y522S/WT^ muscles compared to WT, but significantly lowered by NAC (Figures 5(b) and 5(c)).

Rescue of muscle damage by the *long-term* NAC treatment was also accompanied by functional improvements (Figures 5(d), 5(e), 5(f), and 5(g)). Indeed, although grip strength from 4 to 10 months of age show a slight decrease in all groups of animals (Figure 5(d)), the time-dependent decay of strength was significantly more pronounced in RYR1^Y522S/WT^ mice (~−30%), compared to both WT (~−10%) and NAC-treated RYR1^Y522S/WT^ (~−10%) mice (Figure 5(e)). Finally, *long-term* NAC treatment was also effective in restoring the kinetic-contractility properties (i.e., force frequency and tetanic force) of isolated EDL muscles from RYR1^Y522S/WT^ mice, which were weaker than WT when untreated (Figures 5(f) and 5(g) and Supplementary Figure S2 B).

### 3.5. NAC Treatment Reduces 3-Nitrotyrosine (3-NT) and SOD2 Levels in Skeletal Muscle from RYR1^Y522S/WT^ Mice

**Figure 6:**
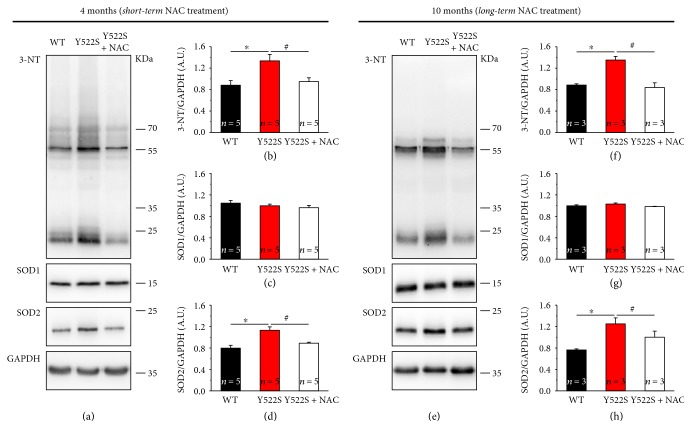
Oxidative stress markers at 4 and 10 months of age following either *short-* or *long-term* treatment. (a and e) Representative immunoblots showing expression of 3-Nitrotyrosine (3-NT), SOD1, and SOD2 in total hind limb muscle homogenates. (b–d) and (f–h) Relative band densities normalized to GAPDH levels. Data are given as mean ± SEM; ^∗^*p* < 0.05, WT versus RYR1^Y522S/WT^ mice; ^#^*p* < 0.05, untreated RYR1^Y522S/WT^ versus NAC-treated RYR1^Y522S/WT^ mice. *n* = number of mice.

We measured in WT, and in untreated or NAC-treated RYR1^Y522S/WT^ mice (both at 4 and 10 months of age), (i) levels of 3-Nitrotyrosine (3-NT), a product of nitration of tyrosine residues of proteins mediated by RNS such as peroxynitrite anion and nitrogen dioxide, which are indicators of oxidative protein damage and inflammation [31–34]; (ii) expression levels of superoxide dismutase types 1 and 2 (SOD1 and SOD2), the two main intracellular enzymes, respectively, localized in the cytoplasm and within mitochondrial matrix, that catalyze the dismutation of anion (O_2_^•−^) into O_2_ and hydrogen peroxide (H_2_O_2_) [35–37], the first step in the elimination of reactive species of oxygen (ROS). WB analyses (Figure 6(a and e)), performed on total hind limb muscle homogenates, revealed that the amount of 3-NT was significantly higher in RYR1^Y522S/WT^ compared to WT mice, with a ~1.5-fold increase (Figure 6(b and f)). NAC treatment normalized 3-NT level to values similar to those of WT mice (Figure 6(a, b, e, and f)). In the same samples, we also measured SOD1 and SOD2 protein contents (Figure 6(a and e)): while there were no differences in SOD1 levels among the three groups of mice (Figure 6(a, c, e, and g)), SOD2 in RYR1^Y522S/WT^ muscles was ~1.5 times higher than that in WT muscles (Figure 6(a, d, e, and h)); also, in this case, NAC treatment brought back SOD2 levels to values closer to those of WT (Figure 6(a, d, e, and h)).

## 4. Discussion

The substitution of a tyrosine with a serine in position 522 (Y522S) of RYR1 results in gain-of-function of the SR Ca^2+^ release channel linked, in humans, to MH with formation of *cores* [10]. The expression of this mutation in an animal model successfully reproduced the human phenotype, as heterozygous RYR1^Y522S/WT^ knock-in mice are MH susceptible [15] and develop structural abnormalities resembling human CCD [17]. The formation of *unstructured* and *contracture cores* in RYR1^Y522S/WT^ muscle fibers is initiated by mitochondrial damage, with abnormalities that then extend to sarcotubular system and contractile elements [17]. The mechanisms linking the RYR1 mutation to the mitochondrial damage in RYR1^Y522S/WT^ muscle fibers are still not fully understood: one possibility is that Ca^2+^-dependant overproduction of ROS and RNS plays a direct role in the destruction of mitochondria [16, 17].

### 4.1. Main Findings of the Present Paper: NAC Ameliorates Structure and Function of RYR1^Y522S/WT^ Muscle by Reducing Oxidative Stress

NAC was previously used to reduce oxidative stress and normalize SR Ca^2+^ release in RYR1^Y522S/WT^ mice and in [16] mice lacking calsequestrin-1 (CASQ1-null) [19, 38]. NAC treatment also reduced the rate of mortality [19] and prevented mitochondrial damage [39] in CASQ1-null mice that, similarly to RYR1^Y522S/WT^ mice, triggers lethal MH episodes when exposed to halogenated anesthetics and heat [40, 41]. The results of the present paper demonstrate that NAC treatment in RYR1^Y522S/WT^ mice (i) reduces formation of *unstructured* and *contracture cores* (Figures 1 and 2) and (ii) improves muscle function, both *in vivo* (grip strength) and ex vivo during electrical stimulation of isolated EDLs (Figure 3). The beneficial effect of NAC treatment on structure/function of RYR1^Y522S/WT^ muscles is evident both when mice were treated starting at 2 months of age, that is, when mitochondrial and fiber damage was still minimal, but also in mice treated for extended periods (6 months of NAC administration; Figure 5) starting at 4 months of age when structural damage was already at an advanced stage, as quantitative analysis in Figure 4 points to significant protection from mitochondrial damage. The reduced formation of *cores* in NAC-treated RYR1^Y522S/WT^ mice could result from the beneficial effect that this treatment exerts on mitochondrial morphology (Figure 4), as we have previously shown that mitochondrial damage plays a pivotal role in the formation of structural *cores* in this mouse model [17].

The role that oxidative stress plays in the events leading to mitochondrial damage and formation of *cores* in CCD has been long discussed. Our present data shows that in NAC-treated RYR1^Y522S/WT^ mice, displaying improved fiber structure/function and reduced mitochondrial damage (Figures 1, 2, 3, 4, and 5), oxidative stress is significantly lower than that in untreated RYR1^Y522S/WT^ mice (Figure 6). In line with these data, we have previously shown that treatment with antioxidants (NAC and Trolox) also lowered oxidative stress in mice with a similar phenotype [19, 39]. Specifically, levels of 3-NT and SOD2, but not of SOD1, which were significantly increased in RYR1^Y522S/WT^ muscles, are reduced to levels more similar to WT following both *short-* and *long-term* NAC treatments (Figure 6). In general, the augmented expression of SOD1 and SOD2 likely reflects a compensatory response to excessive production of ROS [42], a finding in agreement with the observation that the expression of both isoforms increased under different physiopathological conditions in which oxidative stress is elevated [23, 43–46]. The fact that the SOD2 isoform, but not SOD1, is upregulated (Figure 6) suggests that (a) the O_2_^•−^ generated in the mitochondrial matrix plays a central role in the elevated oxidative stress damaging mitochondria and fibers in RYR1^Y522S/WT^ muscles [18] and (b) the effect of mitochondrial-targeted antioxidants should be tested in future studies.

### 4.2. Possible Molecular Mechanisms Linking Excessive SR Ca^2+^ Leak to Oxidative Stress, Mitochondrial Damage, and Formation of Cores

In RYR1^Y522S/WT^ fibers, myoplasmic Ca^2+^ and oxidative stress are both chronically elevated [16, 18]. The Y522S gain-of-function mutation is directly responsible of excessive SR Ca^2+^ leak, whereas the elevated oxidative stress could result from (a) excessive mitochondrial Ca^2+^ uptake, which is known to stimulate the aerobic metabolism [47, 48] and/or (b) increased ATP demand due to the constant need of actively removing excessive myoplasmic Ca^2+^ (by ATP-dependent reuptake into the SR or extrusion in the extracellular space). In turn, Ca^2+^ leak and oxidative stress are linked together in a loop where ROS/RNS results in oxidative modifications of RYR1 channel that enhances the opening probability of the channel and, thus, further Ca^2+^ release from the SR [16]. The accumulation of myoplasmic Ca^2+^ could also result from ROS/RNS-dependent decrease in the activity of both sarcoplasmic/endoplasmic reticulum Ca^2+^ ATPase (SERCA) [49] and plasma membrane Ca^2+^-ATPase (PMCA). Although mitochondrial Ca^2+^ uptake in RYR1^Y522S/WT^ fibers has not been directly measured, chronic elevation of myoplasmic Ca^2+^ could likely result in mitochondrial Ca^2+^ overload, loss of mitochondrial membrane potential, and swelling [50, 51]. The precise disposition of mitochondria next to CRUs (which contain RYR1) in skeletal muscle fibers [20], and their cross talk-based Ca^2+^ signaling [52–56], places these organelles in the unfortunate location to be directly exposed to the excessive Ca^2+^ leak through the mutated RYR1-Y522S channel. In ongoing experiments, we are currently investigating mitochondrial Ca^2+^ uptake in RYR1^Y522S/WT^ fibers.

Chronic Ca^2+^ elevation and ROS/RNS overproduction are likely key players also in proteolysis of contractile elements and oxidation of proteins and lipids of intracellular organelles, such as CRUs and mitochondria. Increased intracellular Ca^2+^ concentration has been linked to muscle damage, mitochondrial swelling, and degeneration of myofibrils, [57–59] and activation of calpains, one of the most important nonlysosomal classes of proteases in skeletal muscle fibers, is indeed activated by increased Ca^2+^ levels [60–62] and by excessive oxidative stress [34, 63, 64]. Activation of calpains has been shown to promote degradation of specific sarcomeric proteins such as titin [24] and disruption of myofibrils [34, 65, 66]. We recently reported that RYR1^Y522S/WT^ and CASQ1-null mice, both having altered Ca^2+^ handling and elevated oxidative stress, displayed levels of calpain activity significantly higher than WT [67]. Consistent with these data, results of the present study indicate that calpain activity is significantly elevated in RYR1^Y522S/WT^ muscles, suggesting that the activation of this proteolytic system could contribute to the disruption of membrane organelles and myofibrillar architecture during formation of *cores*. In support of this hypothesis, our data showed that NAC treatment reduces calpain levels, formation of structural *cores*, and mitochondrial damage (Figures 1, 2, and 4).

### 4.3. Conclusions

The mechanism linking mutations in RYR1 to mitochondrial damage (and consequent formation of structural *cores*) in human CCD has been long discussed and still far from being unraveled. Our work provides some additional insights in the pathogenic mechanisms that underlie mitochondrial damage/disappearance in *cores* of RYR1^Y522S/WT^ mice, supporting the idea that SR Ca^2+^ leak and oxidative stress do play a central role in these events. In addition, our results suggest that NAC administration (or more generally treatment with antioxidants) could be taken into consideration as a long-term therapeutic intervention to reduce the development of *cores* and improve muscle function in patients affected by CCD.

## Supplementary Material

Table S1. This table contains the data plotted in Fig. 1 D. Data are given as mean ± SEM and are expressed as % of total number of fibers analyzed; ∗p<0.05, WT vs. RYR1^Y522S/WT^ mice; ^#^p<0.05, untreated RYR1^Y522S/WT^ vs. NAC-treated RYR1^Y522S/WT^ mice. Table S2. This table contains data plotted in Fig. 4 D-G. Data are given as mean ± SEM; ∗p<0.05, WT vs. RYR1^Y522S/WT^ mice; ^#^p<0.05, untreated RYR1^Y522S/WT^ vs. NAC-treated RYR1^Y522S/WT^ mice. In columns A and C, n = number of measurements; in columns B and D, n = number of fibers analyzed. Table S3. This table contains data plotted in Fig. S1 D-F. Data are given as mean ± SEM; ∗p<0.05, WT vs. RYR1^Y522S/WT^ mice; ^#^p<0.05, untreated RYR1^Y522S/WT^ vs. NAC-treated RYR1^Y522S/WT^ mice. Table S4. This table contains data plotted in Fig. 5 A. Data are given as mean ± SEM and are expressed as % of total number of fibers analyzed; ∗p<0.05, WT vs. RYR1^Y522S/WT^ mice; ^#^p<0.05, untreated RYR1^Y522S/WT^ vs. NAC-treated RYR1^Y522S/WT^ mice. Figure S1. Analysis of proper association between mitochondria and CRUs by EM at 4 months of age. A) Representative EM image showing the association of a mitochondrion with a -CRU. B-D) Bar plots showing the average number / area of EM section of: mitochondria (panel B); CRUs (panel C); and D) mitochondria-CRU pairs (panel C). See also Table S3. Data are given as mean ± SEM; ∗p<0.05, WT vs. RYR1^Y522S/WT^ mice; ^#^p<0.05, untreated RYR1^Y522S/WT^ vs. NAC-treated RYR1^Y522S/WT^ mice. In B-D, n = number of measurements. *Scale bar:* 0.1 μm. Figure S2. Analysis of relationship between relative force (% of maximum) and frequency of stimulation at 4 and 10 months of age. Force-frequency relationship curves of EDL muscles excised from 4 months old (panel A) and 10 months old (panel B) mice in which peak force at each stimulation frequency was normalized to the maximum peak force (120-Hz). Data are given as mean ± SEM. ∗p<0.05, WT vs. RYR1^Y522S/WT^ mice; ^#^p<0.05, untreated RYR1^Y522S/WT^ vs. NAC-treated RYR1^Y522S/WT^ mice.

## Abbreviations

CCD: Central core disease
CM: Confocal microscopy
CRU: Calcium release unit
EC-coupling: Excitation-contraction coupling
EDL: Extensor digitorum brevis
EM: Electron microscopy
NAC: N-acetylcysteine
ROS and RNS: Reactive oxygen and nitrogen species
RYR: Ryanodine receptor
SR: Sarcoplasmic reticulum
TT: Transverse tubules
WT: Wild type.
