# Supplementary material for: Antioxidant Treatment Reduces Formation of Structural Cores and Improves Muscle Function in RYR1Y522S/WT Mice
Source: Oxid Med Cell Longev. 2017 Sep 10;2017:6792694. doi: 10.1155/2017/6792694 (PMC5610828; doi:10.1155/2017/6792694)
Supplement: Supplementary file 1 — Table S1. This table contains the data plotted in Fig. 1 D. Data are given as mean ± SEM and are expressed as % of total number of fibers analyzed; ∗p<0.05, WT vs. RYR1Y522S/WT mice; #p<0.05, untreated RYR1Y522S/WT vs. NAC-treated RYR1Y522S/WT mice. Table S2. This table contains data plotted in Fig. 4 D-G. Data are given as mean ± SEM; ∗p<0.05, WT vs. RYR1Y522S/WT mice; #p<0.05, untreated RYR1Y522S/WT vs. NAC-treated RYR1Y522S/WT mice. In columns A and C, n = number of measurements; in columns B and D, n = number of fibers analyzed. Table S3. This table contains data plotted in Fig. S1 D-F. Data are given as mean ± SEM; ∗p<0.05, WT vs. RYR1Y522S/WT mice; #p<0.05, untreated RYR1Y522S/WT vs. NAC-treated RYR1Y522S/WT mice. Table S4. This table contains data plotted in Fig. 5 A. Data are given as mean ± SEM and are expressed as % of total number of fibers analyzed; ∗p<0.05, WT vs. RYR1Y522S/WT mice; #p<0.05, untreated RYR1Y522S/WT vs. NAC-treated RYR1Y522S/WT mice. Figure S1. Analysis of proper association between mitochondria and CRUs by EM at 4 months of age. A) Representative EM image showing the association of a mitochondrion with a -CRU. B-D) Bar plots showing the average number / area of EM section of: mitochondria (panel B); CRUs (panel C); and D) mitochondria-CRU pairs (panel C). See also Table S3. Data are given as mean ± SEM; ∗p<0.05, WT vs. RYR1Y522S/WT mice; #p<0.05, untreated RYR1Y522S/WT vs. NAC-treated RYR1Y522S/WT mice. In B-D, n = number of measurements. Scale bar: 0.1 μm. Figure S2. Analysis of relationship between relative force (% of maximum) and frequency of stimulation at 4 and 10 months of age. Force-frequency relationship curves of EDL muscles excised from 4 months old (panel A) and 10 months old (panel B) mice in which peak force at each stimulation frequency was normalized to the maximum peak force (120-Hz). Data are given as mean ± SEM. ∗p<0.05, WT vs. RYR1Y522S/WT mice; #p<0.05, untreated RYR1Y522S/WT vs. NAC-treated RYR1Y522S/WT mice. [file 6792694.f1.docx]

SUPPLEMETARY TABLES and FIGURES

|  | A | B | C |
| --- | --- | --- | --- |
|  | Normal fibers | Fibers with *unstructured* *cores* | Fibers with *contracture* *cores* |
| WT  (3 mice, 90 fibers analyzed) | 100 ± 0 | 0 | 0 |
| RYR1^Y522S/WT^  (3 mice, 91 fibers analyzed) | *63 ± 1 | *14 ± 1 | *23 ± 3 |
| RYR1^Y522S/WT^+NAC  (3 mice, 98 fibers analyzed) | ^#^92 ± 2 | ^#^6 ± 1 | ^#^3 ± 1 |

Table S1. This table contains the data plotted in Fig. 1 D. Data are given as mean ± SEM and are expressed as % of total number of fibers analyzed; *p<0.05, WT vs. RYR1^Y522S/WT^ mice; ^#^p<0.05, untreated RYR1^Y522S/WT^ vs. NAC-treated RYR1^Y522S/WT^ mice.

|  | A | B | C | D |
| --- | --- | --- | --- | --- |
|  | Damaged mitochondria,  % | Average n. of damaged mitochondria  /100 μm^2^ | Size of apparently normal mitochondria,  nm^2^ x10^3^ | Mitochondrial volume/total volume,  % |
| WT  (3 mice) | 5  (n= 457) | 1.9 ± 0.2  (n= 30) | 63.5 ± 0.7  (n= 1522) | 3.4 ± 0.2  (n= 30) |
| RYR1^Y522S/WT^  (3 mice) | *23  (n= 1046) | *4.3 ± 0.4  (n= 30) | *103.2 ± 2.8  (n= 571) | *6.2 ± 0.3  (n= 30) |
| RYR1^Y522S/WT^+NAC  (3 mice) | ^#^13  (n= 1151) | ^#^2.3 ± 0.3  (n= 30) | ^#^78.6 ± 1.6  (n=832) | 5.0 ± 0.2  (n= 30) |

Table S2. This table contains data plotted in Fig. 4 D-G. Data are given as mean ± SEM; *p<0.05, WT vs. RYR1^Y522S/WT^ mice; ^#^p<0.05, untreated RYR1^Y522S/WT^ vs. NAC-treated RYR1^Y522S/WT^ mice. In columns A and C, n = number of measurements; in columns B and D, n = number of fibers analyzed.

|  | A | B | C |
| --- | --- | --- | --- |
|  | Mitochondrial number/100 μm^2^ | CRUs number  /100 μm^2^ | MITO-CRUs pairs  /100 μm^2^ |
| WT  (3 mice, 20 fibers analyzed) | 34.2 ± 0.9 | 51.0 ± 1.2 | 25.4 ± 0.7 |
| RYR1^Y522S/WT^  (3 mice, 18 fibers analyzed) | *25.9 ± 0.7 | *42.4 ± 1.0 | *11.9 ± 0.5 |
| RYR1^Y522S/WT^ + NAC  (3 mice, 30 fibers analyzed) | ^#^29.1 ± 0.6 | 36.5 ± 1.1 | ^#^14.6 ± 0.5 |

Table S3. This table contains data plotted in Fig. S1 D-F. Data are given as mean ± SEM; *p<0.05, WT vs. RYR1^Y522S/WT^ mice; ^#^p<0.05, untreated RYR1^Y522S/WT^ vs. NAC-treated RYR1^Y522S/WT^ mice.

|  | A | B | C |
| --- | --- | --- | --- |
|  | Normal fibers | Fibers with *unstructured* *cores* | Fibers with *contracture* *cores* |
| WT  (3 mice, 90 fibers analyzed) | 98 ± 1 | 0 | 2 ± 1 |
| RYR1^Y522S/WT^  (3 mice, 91 fibers analyzed) | *48 ± 5 | *18 ± 2 | *30 ± 4 |
| RYR1^Y522S/WT^+NAC  (3 mice, 98 fibers analyzed) | ^#^77 ± 4 | ^#^10 ± 2 | ^#^13 ± 2 |

Table S4. This table contains data plotted in Fig. 5 A. Data are given as mean ± SEM and are expressed as % of total number of fibers analyzed; *p<0.05, WT vs. RYR1^Y522S/WT^ mice; ^#^p<0.05, untreated RYR1^Y522S/WT^ vs. NAC-treated RYR1^Y522S/WT^ mice.

***
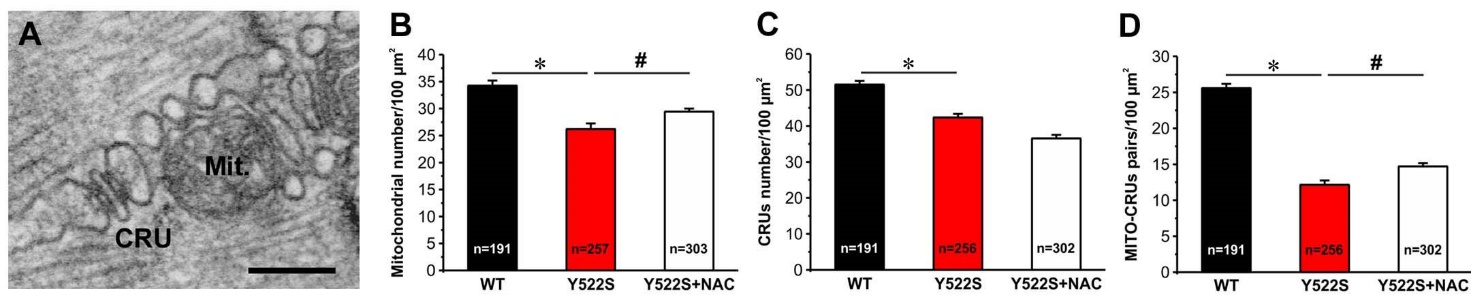
***

Figure S1. Analysis of proper association between mitochondria and CRUs by EM at 4 months of age. A) Representative EM image showing the association of a mitochondrion with a -CRU. B-D) Bar plots showing the average number / area of EM section of: mitochondria (panel B); CRUs (panel C); and D) mitochondria-CRU pairs (panel C). See also Table S3. Data are given as mean ± SEM; *p<0.05, WT vs. RYR1^Y522S/WT^ mice; ^#^p<0.05, untreated RYR1^Y522S/WT^ vs. NAC-treated RYR1^Y522S/WT^ mice. In B-D, n = number of measurements. *Scale bar:* 0.1 µm.


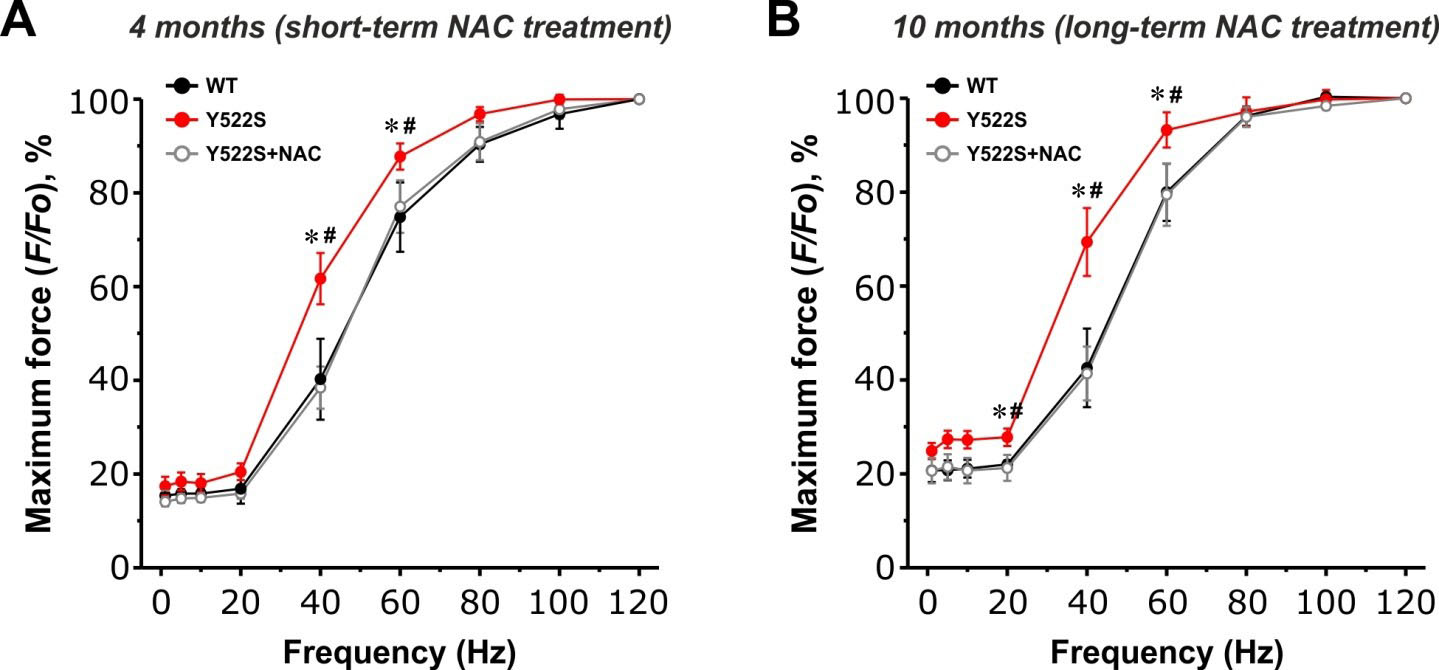


Figure S2. Analysis of relationship between relative force (% of maximum) and frequency of stimulation at 4 and 10 months of age. Force-frequency relationship curves of EDL muscles excised from 4 months old (panel A) and 10 months old (panel B) mice in which peak force at each stimulation frequency was normalized to the maximum peak force (120-Hz). Data are given as mean ± SEM. *p<0.05, WT vs. RYR1^Y522S/WT^ mice; ^#^p<0.05, untreated RYR1^Y522S/WT^ vs. NAC-treated RYR1^Y522S/WT^ mice.
